# Transforming Growth Factor β Inhibits Platelet Derived Growth Factor-Induced Vascular Smooth Muscle Cell Proliferation via Akt-Independent, Smad-Mediated Cyclin D1 Downregulation

**DOI:** 10.1371/journal.pone.0079657

**Published:** 2013-11-13

**Authors:** Abel Martin-Garrido, Holly C. Williams, Minyoung Lee, Bonnie Seidel-Rogol, Xinpei Ci, Jin-Tang Dong, Bernard Lassègue, Alejandra San Martín, Kathy K. Griendling

**Affiliations:** 1 Department of Medicine, Division of Cardiology, Emory University, Atlanta, Georgia, United States of America; 2 Winship Cancer Institute, Department of Hematology and Medical Oncology, Emory University, Atlanta, Georgia, United States of America; Osaka University Graduate School of Medicine, Japan

## Abstract

In adult tissue, vascular smooth muscle cells (VSMCs) exist in a differentiated phenotype, which is defined by the expression of contractile proteins and lack of proliferation. After vascular injury, VSMC adopt a synthetic phenotype associated with proliferation, migration and matrix secretion. The transition between phenotypes is a consequence of the extracellular environment, and in particular, is regulated by agonists such as the pro-differentiating cytokine transforming growth factor β (TGFβ) and the pro-proliferative cytokine platelet derived growth factor (PDGF). In this study, we investigated the interplay between TGFβ and PDGF with respect to their ability to regulate VSMC proliferation. Stimulation of human aortic VSMC with TGFβ completely blocked proliferation induced by all isoforms of PDGF, as measured by DNA synthesis and total cell number. Mechanistically, PDGF-induced Cyclin D1 mRNA and protein expression was inhibited by TGFβ. TGFβ had no effect on PDGF activation of its receptor and ERK1/2, but inhibited Akt activation. However, constitutively active Akt did not reverse the inhibitory effect of TGFβ on Cyclin D1 expression even though inhibition of the proteasome blocked the effect of TGFβ. siRNA against Smad4 completely reversed the inhibitory effect of TGFβ on PDGF-induced Cyclin D1 expression and restored proliferation in response to PDGF. Moreover, siRNA against KLF5 prevented Cyclin D1 upregulation by PDGF and overexpression of KLF5 partially reversed TGFβ-induced inhibition of Cyclin D1 expression. Taken together, our results demonstrate that KLF5 is required for PDGF-induced Cyclin D1 expression, which is inhibited by TGFβ via a Smad dependent mechanism, resulting in arrest of VSMCs in the G1 phase of the cell cycle.

## Introduction

Plasticity of vascular smooth muscle cells (VSMC) from adult tissue is an important component of proliferative vascular diseases. Normally, VSMCs exhibit a differentiated phenotype, defined by the expression of contractile proteins such as smooth muscle alpha actin (SMA), calponin and smooth muscle heavy chain [1]. However, in response to external signals, VSMCs can modify their phenotype and become synthetic, proliferative and migratory. This switch from contractile (non-proliferative) to synthetic (proliferative) phenotype occurs in different cardiovascular diseases such as atherosclerosis and restenosis [1], thus contributing to lesion formation. Strategies to prevent transition to the proliferative phenotype or to revert cells to the contractile phenotype are therefore attractive therapeutic options.

Transforming growth factor β (TGFβ) has been shown to be a potent inducer of smooth muscle specific differentiation gene expression. It exerts its effects by binding to and dimerizing TGFβ receptor (TGFβR) type I and type II. After dimerization, the kinase activity of TGFβR increases the phosphorylation of several targets, which can be divided into Smad-dependent and non-Smad-dependent pathways (reviewed in [2]). In the Smad pathway, activation of TGFβR leads to the phosphorylation of Smad 2 and 3, which then complex with Smad4 to translocate to the nucleus and mediate gene expression. Recently, non-Smad pathways, which include Akt, p38 mitogen-activated protein kinase, extracellular signal regulated kinase (ERK) and c-Jun N-terminal kinase (JNK), have also been shown to be important for the full activity TGFβ (reviewed in [3]). Activation of the TGFβR can also lead to stimulation of TGFβ-activated kinase 1 (TAK1) independent of receptor kinase activity but dependent upon receptor dimerization [4]. The complexity of signalling pathways utilized by TGFβ to modulate cellular function makes it challenging to understand which signals underlie particular physiological responses.

In the vasculature, TGFβ has been linked to several processes. Studies in knockout mice deficient in critical elements of TGFβ signalling pathways such as TGFβR type II [5] and Smad4 [6] develop early lethality due to impaired vasculogenesis. Besides its role in development, the best characterized effect of TGFβ in the vasculature is as a pro-differentiation cytokine. Thus, stimulation of TGFβ signaling induces the contractile phenotype characterized by the expression of SMA, calponin and SM-MHC in VSMC [7]–[9]. More controversial is its effect on proliferation. An early study showed that TGFβ is expressed at high levels after vascular injury [10]. In concordance with this study, administration of neutralizing antibody against TGFβ reduces neointimal formation after vascular injury [11], [12]. In contrast to these reports, several laboratories showed that TGFβ can reduce the proliferation of VSMCs in response to mitogens such as serum [13], epidermal growth factor (EGF) [14], [15] and platelet derived growth factor (PDGF) [16]. The antiproliferative effect of TGFβ in VSMCs seems to be Smad-dependent since the decrease of Smad3 levels by small interfering RNA (siRNA) or knockout of Smad4 partially suppresses the antiproliferative effect of TGFβ on serum-induced growth [6], [13]. On the other hand, Majack et al. [16] observed that TGFβ acts a proliferative agent in confluent VSMCs, whereas in sub-confluent cultures it reduces the proliferation induced by serum. Thus, the effect that a particular cytokine like TGFβ has on differentiation/proliferation depends upon complex environmental cues and tight regulation of intracellular signalling pathways.

Cell proliferation is largely regulated by a closely controlled, time-dependent activation of cell cycle proteins. Among them, cyclins play a prominent role because their expression promotes progression through different phases of the cell cycle (reviewed in [17]). In particular, Ruef et al. [18] demonstrated that the administration of flavopiridol reduces neointima formation after vascular injury partially via reduction of Cyclin D1 expression. Cyclin D1 induces progression from phase G1 to S via activation of cyclin-dependent kinase-4 (Cdk4), and its expression correlates with the proliferation of VSMCs [19]–[22]. Expression of Cyclin D1 can be regulated transcriptionally or post transcriptionally. Perhaps the best characterized activator of Cyclin D1 transcription is ERK 1/2, acting via upregulation of the transcription factor AP-1 (reviewed in [23]). Alternatively, pioneering work from Diehl et al. [24], [25] showed that phosphoinositol 3-Kinase/Akt/Glycogen synthase kinase-3β phosphorylates Cyclin D1 on Thr 286, which decreases its stability by increasing proteasomal degradation. Because both of these pathways are activated by TGFβ, the ultimate effect of TGFβ on Cyclin D1 in VSMCs is difficult to predict.

In the vasculature, VSMCs are exposed to a cocktail of cytokines, often including pro- and anti-proliferative hormones and peptides. Therefore, the confluence of signalling pathways activated will determine the ultimate response of the cell. In this study, we sought to define the precise mechanism whereby TGFβ exerts an antiproliferative effect in human VSMCs in the setting of progrowth conditions. Using PDGF-BB as a prototypical mitogen (because of its known role in lesion formation after vascular injury [26], [27]), we report that TGFβ inhibits the proliferation induced by PDGF via Akt-independent, Smad4- and Krueppel-like factor 5 (KLF5)-dependent repression of Cyclin D1 expression.

## Materials and Methods

### Materials

TGFβ1 and PDGF-BB were obtained from R&D Systems (Minneapolis, MN). The following antibodies were used for Western blot: β-tubulin (Sigma, St. Louis, MO); phospho-Akt Ser 473, phospho-Akt Ser 308, Akt, phospho-p44/42 MAPK (Erk1/2) (Thr202/Tyr204), phospho-PDGF Receptor-β Tyr 751 and Smad4 (Cell Signaling Technology, Beverly, MA); Cyclin D1 (A-12) and GAPDH (Santa Cruz, Dallas, TX); KLF5 (EMD Millipore, Billerica, MA). The inhibitor of TGFβ receptor kinase (SB-431542) was purchased from Sigma.

### Cell culture

Human aortic smooth muscle cells from an 18-year-old male donor were obtained from Cascade Biologics (Gibco, Portland, OR). Cells were cultured as recommended by the manufacturer and used between passages 5–8. Cells stained positively for smooth muscle alpha-actin and calponin, confirming their identity as smooth muscle cells.

### Western blot

VSMCs were lysed in Hunter's buffer (25 mM HEPES, 150 mM NaCl, 1.5 mM MgCl_2_, 1 mM EGTA, 10 mM Na-pyrophosphate, 10 mM NaF, 0.1 mM Na-orthovanadate, 1% Na deoxycholate, 1% Triton X-100, 0.1% SDS, 10% Glycerol, and protease inhibitors), as described previously [28]. Proteins were separated using SDS-PAGE and transferred to nitrocellulose membranes, blocked, and incubated with appropriate primary antibodies. Proteins were detected by ECL (Amersham, Sunnyvale, CA). Band intensity was quantified by densitometry using ImageJ 1.38 software.

### RNA isolation and qRT-PCR

Total RNA was purified from cells using the RNeasy kit (Qiagen, Valencia, CA), as recommended by the manufacturer. First-strand cDNA synthesis was performed using 5 µg total RNA per sample, random 15-mer oligonucleotide primers (Sigma) and Superscript II reverse transcriptase (Invitrogen, Grand Island, NY), according to the supplier's instructions. Quantitative PCR was carried out with a LightCycler instrument (Roche Applied Science, Indianapolis, IN) in glass capillaries. Human cyclin D1 cDNA was amplified using predesigned primers (QuantiTect primer assay), annealing at 55°C, and SYBR Green PCR master mix from Qiagen. Expression of cyclin D1 was normalized to the housekeeping gene human TATA box binding protein. Primers for this gene were purchased from Real Time Primers (Elkins Park, PA), forward: 5′-TATAATCCCAAGCGGTTTGC-3′ and reverse: 5′-GCTGGAAAACCCAACTTCTG-3′. Reaction conditions were: 100 mM primers, 3 mM MgCl_2_, annealing at 58°C, using PlatinumTaq DNA polymerase and SYBR green dye from Invitrogen. Quantitative PCR data analysis was performed using the mak3 module of the qPCR software library in the R environment [29]–[31].

### Proliferation assay

Two independent methods were used for the quantification of cell proliferation.

A) Total cell number. Briefly, for experiments on subconfluent cells, 45600 cells were seeded in 12-well plates (Corning) in complete medium. The next day, VSMCs were incubated in serum-free medium for 24 hours prior to stimulation with PDGF at 20 ng/ml and/or TGFβ at 1 ng/ml in serum free medium. After 24 hours, the medium was replaced with fresh cytokines. After 48 hours, VSMCs were trypsinized and the cell number was quantified using a Scepter automated cell counter (Millipore, Billerica, MA). Each experiment was performed in triplicate and each replicate was counted 3 times.

B) DNA quantification. VSMCs (12,000/well) were seeded in 24-well plates (Corning) in complete medium. The next day, they were incubated in serum free medium for 24 hours prior to stimulation. VSMCs were treated with PDGF at 20 ng/ml and/or TGFβ at 1 ng/ml in serum-free medium. Cytokines were replenished in fresh medium at 24 hours, and after 48 hours, DNA was quantified using CyQuant NF (Invitrogen) according to the manufacturer's protocol. Fluorescence intensities of triplicate samples were measured with a fluorescence microplate reader using excitation at 485 nm and fluorescence detection at 530 nm.

### Virus production and VSMC infection

Constitutively active Akt adenovirus (Ad-myrAkt) contains the c-Src myristoylation sequence that targets the fusion protein to the membrane fused in frame to the N-terminus of the HA-tagged Akt (wild type) coding sequence. The control virus, Ad-β-Gal, contains the bacterial β-galactosidase gene downstream from the cytomegalovirus promoter/enhancer. All constructs were amplified in 293 cells and purified by ultracentrifugation in the presence of CsCl. For transfection, VSMCs (70% confluency) were incubated with the adenovirus at a multiplicity of infection (MOI) of 100 in growth medium for 1 hour. Virus was removed when the medium was replaced.

The pSin-flag-KLF5 expression vector was constructed using a PCR approach with the template pcDNA-flag-KLF5 [32]. pSin-mCherry was used as a control. Lentiviruses were prepared and used following the lentiviral protocols described on the Addgene website.

### siRNA studies

VSMCs were transfected with 25 nM small interference RNA against Smad4 (siSmad4) (sense 5′-CAAGGUUGCACAUAGGCAAd(TT)-3′ and antisense 5′-UUGCCUAUGUGCAACCUUGd(CT)-3′), 100 nM small interference RNA against KLF5 (siKLF5) (sense: 5′-AAGCUCACCUGAGGACUCATT-3′ and antisense: 5′-UGAGUCCUCAGGUGAGCUUTT-3′) or with the All-Star negative control siRNA (Qiagen) using siRNA max (Invitrogen) in complete medium. The next day, the medium was replaced with serum-free medium and then cultured in serum-free media for 24 hours prior to treatments.

### Statistical analysis

Results are expressed as mean ± S.E. from at least three independent experiments. Statistical significance was assessed using Student's t-test or analysis of variance (ANOVA), followed by Bonferroni's Multiple Comparison post-hoc test. A value of *p*<0.05 was considered significant.

## Results

### TGFβ inhibits PDGF-induced proliferation of VSMCs

**Figure 1:**
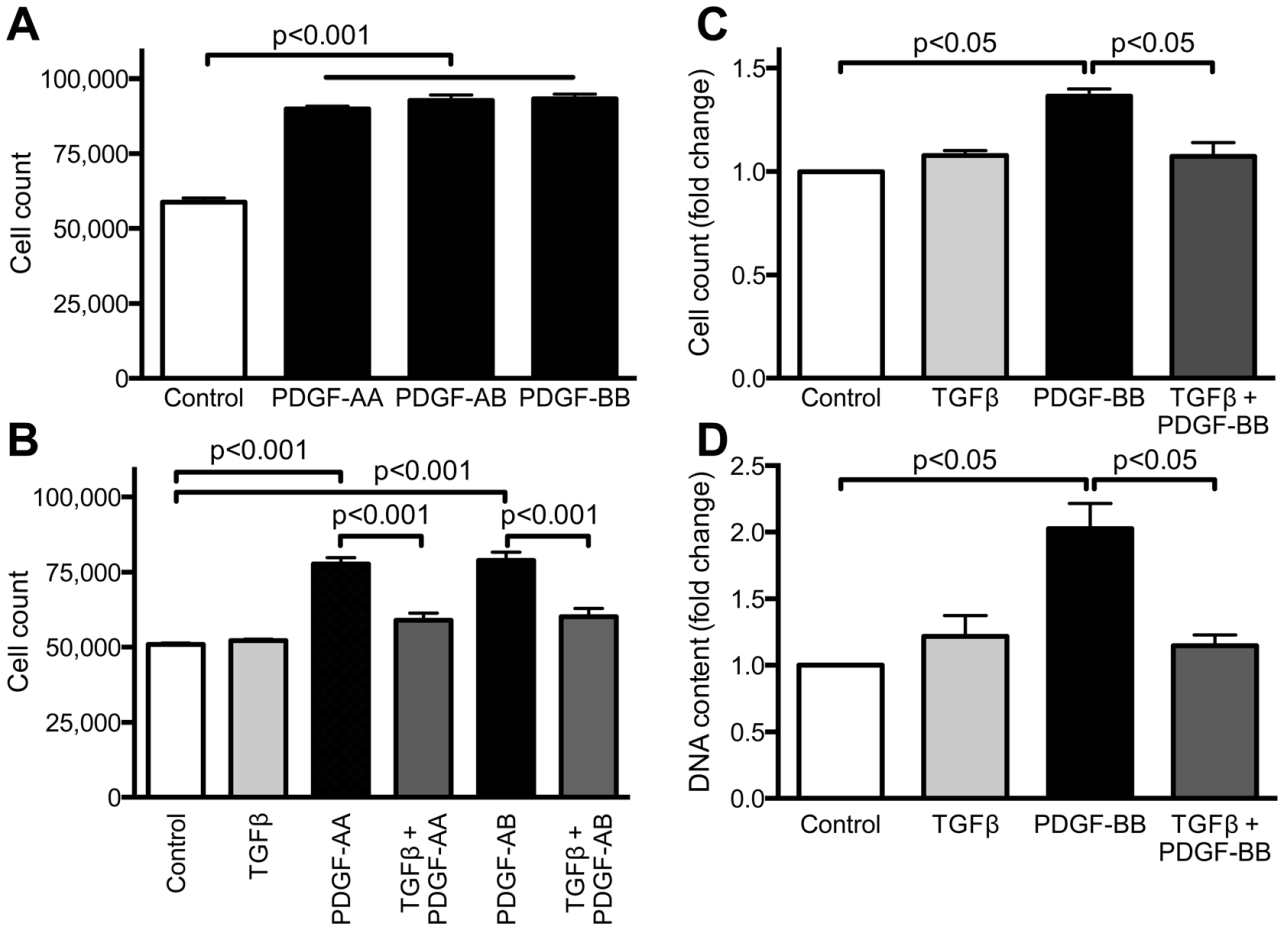
TGFβ inhibits PDGF-induced proliferation. Subconfluent VSMCs were stimulated for 48(20 ng/mL, every 24 h) or TGFβ (1 ng/mL), alone or in combination. A–C. Cells were counted using a Scepter instrument. D. DNA content was measured using the fluorescent indicator CyQuant. Data are expressed as mean ± SE of fold change over control in three independent experiments.

We and others previously showed that PDGF is a potent VSMC proliferative agent [33]–[35]. However, the role of TGFβ in VSMC proliferation is contradictory since it has been reported to increase [16], [36] or decrease VSMC proliferation [13], [16], [37]. Battegay et al. [38] showed that TGFβ can act as a pro-proliferative agent at low concentrations (0.02 ng/ml), while reducing SMC proliferation at high concentrations (1–10 ng/ml). Therefore, we used 1 ng/ml of TGFβ (which is comparable to normal circulating levels of TGFβ [39]), and assessed the effect of TGFβ on VSMC proliferation induced by different isoforms of PDGF. As shown in Fig. 1A, 20 ng/ml PDGF-AA, -AB- and –BB had equivalent effects on proliferation of subconfluent VSMCs, as measured by cell counts after 48 h. TGFβ (1 ng/mL) added in combination with any of the isoforms of PDGF nearly abolishes this increase in cell number (Fig. 1B–C). Similar effects are not observed when serum, rather than PDGF, is used as the growth stimulus (serum 2.1±0.2% control; serum + TGFβ 2.0±0.3% control). Because the responses to all three types of PDGF were similar and TGFβ was equally effective in inhibiting growth in all three conditions, PDGF-BB (designated PDGF from here on) was used for further experiments.

To verify that this increase in cell number is due to cell cycle progression, we measured DNA content of VSMCs stimulated with PDGF, TGFβ or both. Our results show that stimulation with PDGF, but not TGFβ, increases DNA content (2.0±0.2-fold increase, PDGF vs. control), but more importantly, co-stimulation with TGFβ and PDGF completely inhibits the PDGF-induced increase in DNA content in VSMC (1.1±0.1-fold increase vs control) (Fig. 1D). Finally, to determine if confluency affected the ability of TGFβ to inhibit PDGF-BB-induced proliferation, we plated cells at near confluent densities, and stimulated with PDGF, TGFβ or both. TGFβ retained its ability to block proliferation (data not shown).

### TGFβ inhibits PDGF induction of Cyclin D1 via receptor-dependent kinase activity

**Figure 2:**
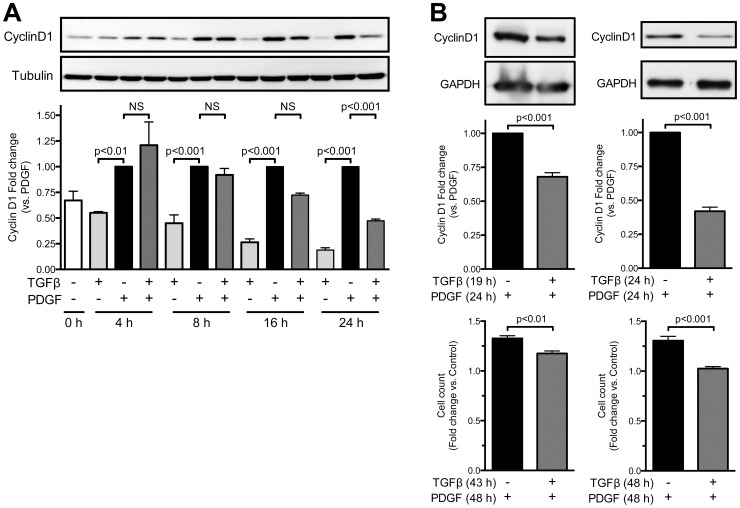
TGFβ inhibition of PDGF-induced CyclinD1 expression is time-dependent. A. Subconfluent VSMCs were stimulated for the indicated times with PDGF-BB (20 ng/mL, every 24 h) or TGFβ (1 ng/mL), alone or in combination. Cyclin D1 expression was determined by western blot. Tubulin was used as a loading control. Data were normalized to tubulin and are expressed as mean ± SE of fold change from PDGF stimulation from 3 independent experiments. B. Subconfluent VSMCs were stimulated with PDGF-BB (20 ng/mL) for 5 h prior to addition of TGFβ (1 ng/mL) for the remaining 24 h (for Cyclin D1 expression) or 48 h (for cell growth). Cyclin D1 expression was determined by western blot, and growth was assessed by cell counting. GAPDH was used as a loading control. Data were normalized to GAPDH for western blot and in both cases are expressed as mean ± SE of fold change (vs. PDGF for Cyclin D1 and vs. control for cell growth) from 3 independent experiments.

Fig. 1 shows that TGFβ inhibits PDGF-induced proliferation prior to stimulation of DNA synthesis (S phase). Previous studies have demonstrated that expression of Cyclin D1 is critical for the progression of the cell cycle from G1 to S phase (reviewed in [23]). We therefore hypothesized that TGFβ might regulate the expression of Cyclin D1 induced by PDGF. Cells were exposed to PDGF, TGFβ or the combination for 4–24 hours, and Cyclin D1 expression was measured by western blot. TGFβ-induced inhibition of the increase in Cyclin D1 in response to PDGF was apparent at 16 h and statistically significant at 24 h (Fig. 2A). However, when TGFβ is added 5 hours after PDGF, cyclin D1 is still significantly downregulated and growth is inhibited, albeit to a lesser extent (Fig 2B).

**Figure 3:**
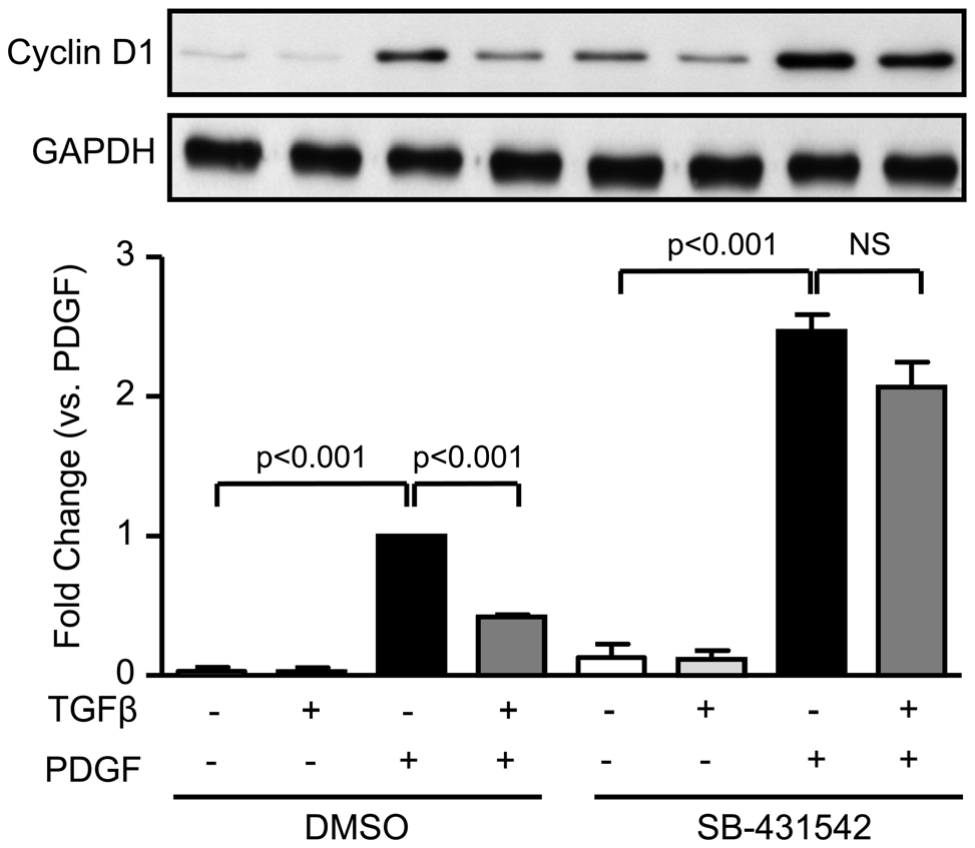
TGFβ inhibits PDGF-induced CyclinD1 expression via TGFR. VSMCs were stimulated for 24-BB (20 ng/mL), or TGFβ (1 ng/mL), alone or in combination. In some experiments, SB431542 (10 µM) was added to the cells 90 minutes prior to stimulation with PDGF or TGFβ. Cyclin D1 expression was determined by western blot. GAPDH is used as a loading control. Data were normalized to GAPDH and are expressed as mean ± SE of fold change from PDGF stimulation in 3 independent experiments.

Because signalling through the TGFβ receptor can be kinase activity dependent or independent [4], we pre-incubated cells with SB-431542, a specific TGFβR kinase inhibitor, before stimulation with PDGF alone or in combination with TGFβ. We found that TGFβR kinase activity is required for the inhibition of PDGF-induced Cyclin D1 expression by TGFβ since incubation of VSMC with SB-431542 reversed the inhibitory effect of TGFβ on Cyclin D1 protein levels (Fig. 3). In fact, the response to PDGF was significantly augmented even in the absence of TGFβ (p<0.001), suggesting that the cells themselves produce TGFβ that activates the TGFβR and blunts the response to exogenous PDGF.

### TGFβ inhibits PDGF-induced Cyclin D1 expression via an Akt-independent mechanism

**Figure 4:**
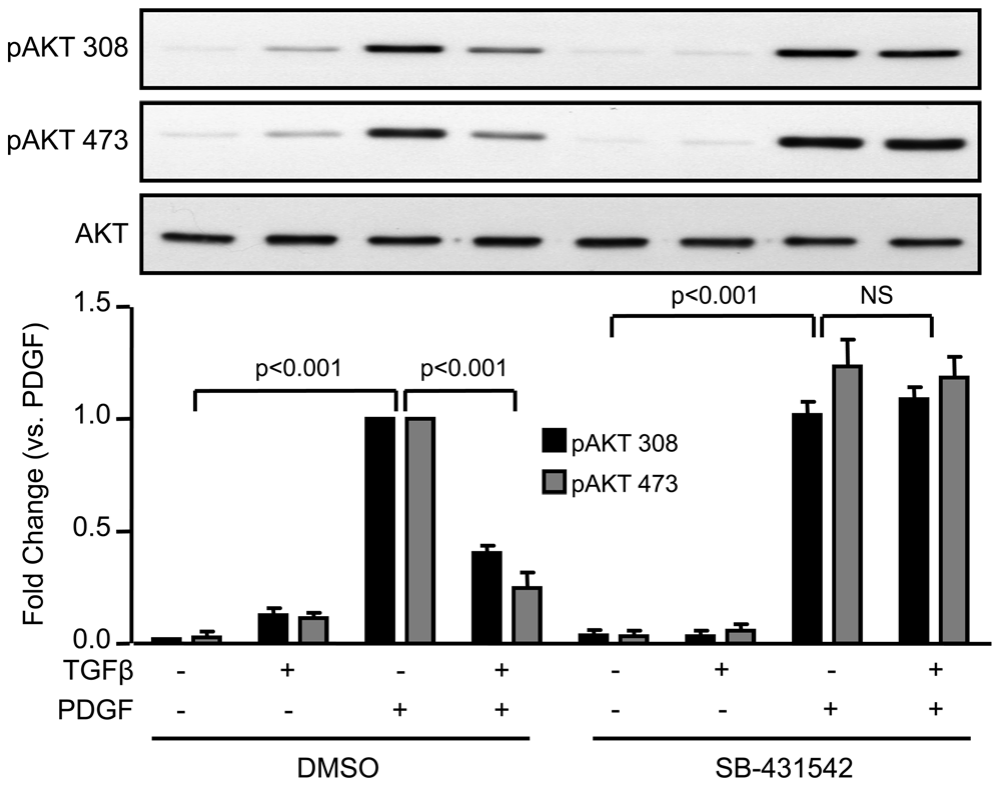
TGFβ inhibits PDGF-induced Akt activation via TGFR. VSMCs were treated as described in Fig. 1, lysed and prepared for western blot. Akt activation was assessed using phosphoantibodies against Thr308 and Ser473. Data were normalized to total Akt and are expressed as mean ± SE of fold change from PDGF stimulation in three independent experiments.

**Figure 5:**
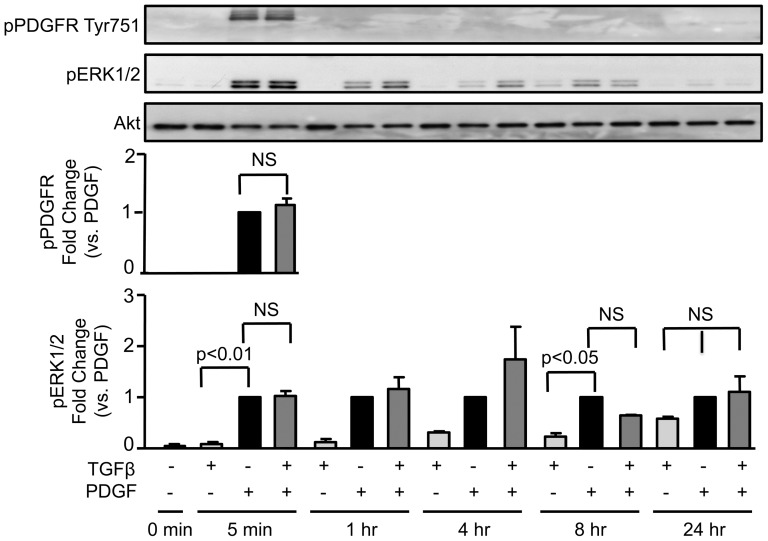
TGFβ does not inhibit PDGFR or ERK activation by PDGF. VSMCs were treated for the indicated times with PDGF-BB (20 ng/mL), TGFβ (1 ng/mL) or both, lysed and analyzed by western blot using phosphospecific antibodies to Tyr 751 of the PDGFR and Thr 202/Tyr 204 of ERK 1/2, respectively. Total Akt is shown as a loading control. Data are expressed as mean ± SE of fold change from PDGF stimulation in three independent experiments. For PDGFR, significance was assessed by t-test; for ERK1/2, significance was assessed as described in Methods.

To determine the mechanism by which TGFβR kinase activation blocks Cyclin D1 induction by PDGF, we focused on signalling pathways previously shown to be upstream of Cyclin D1. PDGF has been shown to activate Akt, which in other cell types, increases Cyclin D1 levels via stabilization of the protein [24], [25]. As expected, PDGF increases Akt activation at 24 hours as measured by western blotting using specific antibodies against phospho-Akt T308 and S473 (Fig. 4). Co-incubation with TGFβ significantly blunts this response (∼60% of reduction in TGFβ + PDGF compared to PDGF alone). In concordance with Fig. 3, inhibition of TGFβR kinase activity reversed the effect of TGFβ on Akt activation induced by PDGF (Fig. 4). Conversely, neither PDGFR phosphorylation nor ERK1/2 activation was altered by TGFβ (Fig. 5), indicating that TGFβ does not block PDGF signalling indiscriminately.

**Figure 6:**
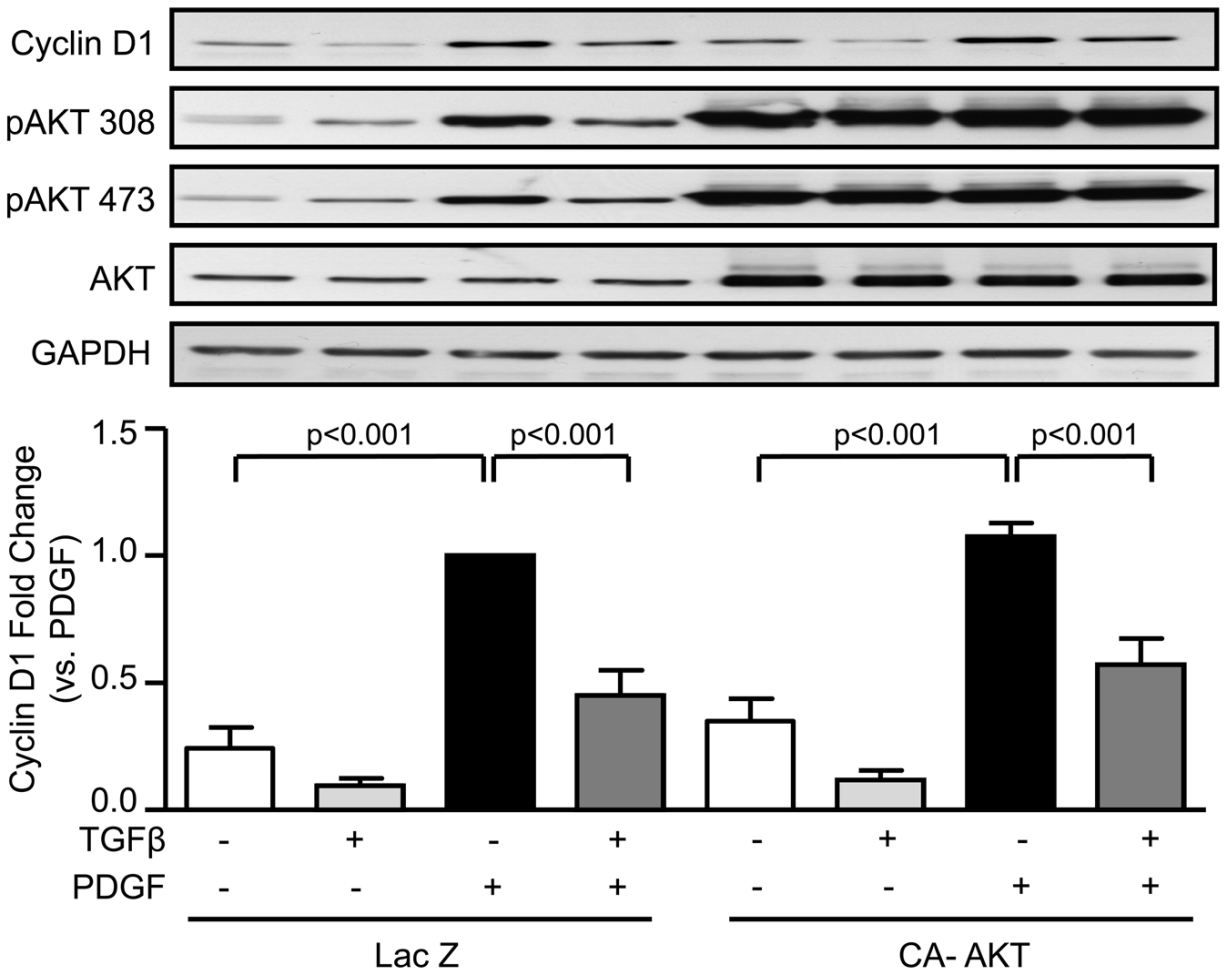
TGFβ-mediated inhibition of PDGF-induced Cyclin D1 expression is Akt-independent. VSMCs were transduced with control adenovirus (LacZ) or an adenovirus expressing constitutively active Akt (CA-Akt) prior to stimulation with PDGF-BB, TGFβ or both as described in Fig. 2. Cyclin D1 expression and Akt activation were determined by western blot. Data were normalized to GAPDH and expressed as mean ± SE of fold change from PDGF stimulation.

To determine whether Akt is important for CyclinD1 expression, VSMCs were transduced with a constitutively active form of Akt (myristolated Akt) to overcome the Akt inhibition and treated with PDGF, TGFβ or both together. Fig. 6 shows that TGFβ is still able to inhibit PDGF-induced CyclinD1 expression in cells overexpressing the active form of Akt, suggesting that reduction of Akt activation is not the pathway by which TGFβ inhibits CyclinD1 expression in response to PDGF.

### TGFβ inhibition of CyclinD1 expression and proliferation is mediated by transcriptional and proteolytic pathways

**Figure 7:**
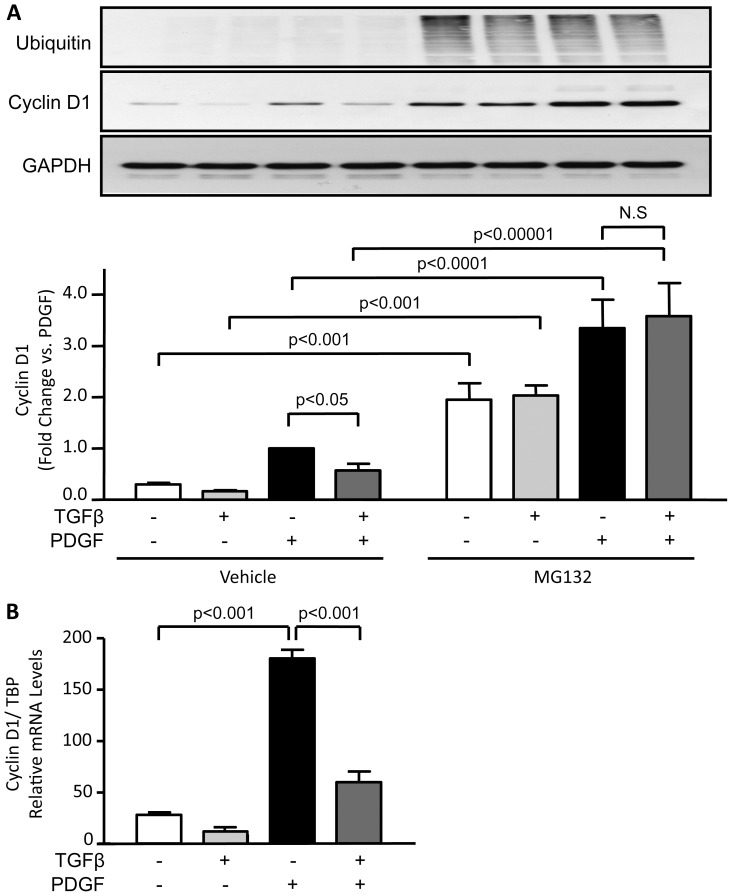
TGFβ-induced downregulation of Cyclin D1 is mediated by both proteasomal degradation and regulation of mRNA. A. VSMCs were stimulated with PDGF-BB (20 ng/mL), or TGFβ (1 ng/mL) for 24 h, alone or in combination. The ubiquitin proteasome inhibitor MG132 (10 µM) was added to the cells 5 h after growth factor stimulation. Cyclin D1 expression was determined by western blot. GAPDH is used as a loading control. Data were normalized to GAPDH and are expressed as mean ± SE of fold change from PDGF stimulation in 4 independent experiments. B. VSMCs were treated with PDGF-BB, TGFβ or both as described in Fig. 1. Cyclin D1 and TATA box binding protein (TBP) mRNA expression were determined by qRT-PCR. Cyclin D1 mRNA levels are expressed relative to TBP mRNA. Values represent mean ± SE from three independent experiments.

Cyclin D1 expression has been shown to be regulated by proteolytic degradation through the proteasome [24], [25]. To determine if proteasomal degradation plays a role in TGFβ-induced Cyclin D1 downregulation, we blocked the proteasome with MG132. As shown in Fig. 7A, TGFβ is no longer able to decrease Cyclin D1 protein levels when the proteasome is inhibited. However, we were unable to detect ubiquitination of Cyclin D1 (data not shown), suggesting that perhaps a transcription factor that regulates Cyclin D1 is regulated by proteasomal degradation. To test this possibility, we first evaluated the levels of CyclinD1 mRNA in VSMCs treated with TGFβ, PDGF or both. Fig. 7B shows that PDGF increases the level of Cyclin D1 mRNA, while TGFβ slightly reduces it. When VSMCs are co-treated with TGFβ and PDGF, the positive effect of PDGF on Cyclin D1 mRNA is completely blunted.

**Figure 8:**
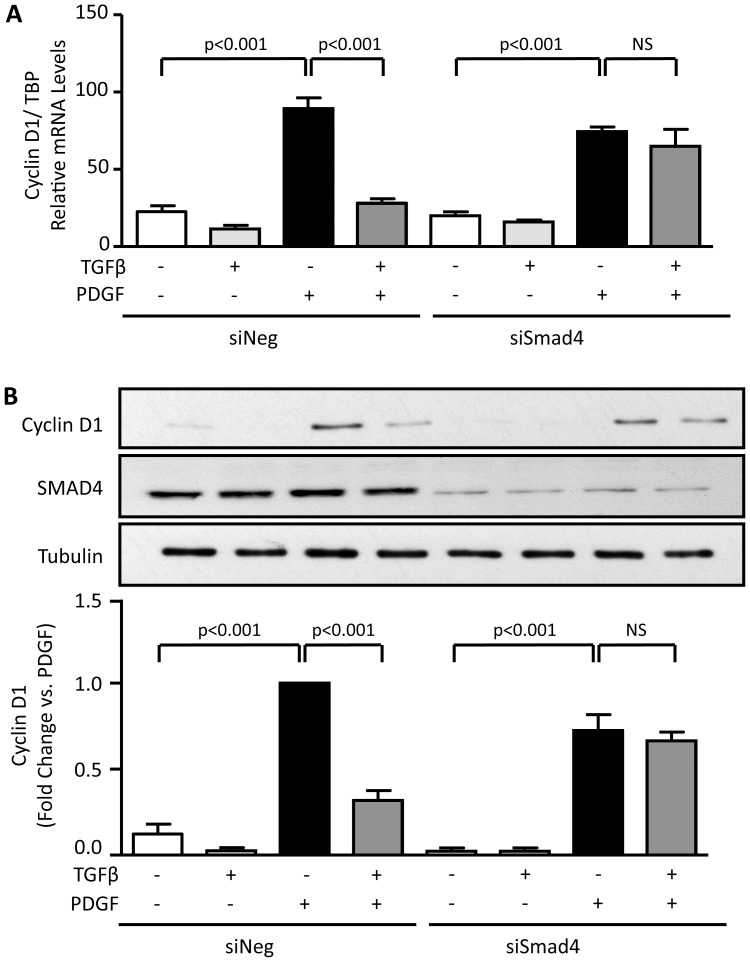
Smad4 is required for TGFβ-mediated inhibition of PDGF-induced CyclinD1 expression. VSMCs were transfected with control siRNA (siNeg) or siRNA against Smad4 (siSmad4) prior to stimulation with PDGF-BB, TGFβ or both as described in Fig. 3. A. Cyclin D1 mRNA was measured by qPCR and normalized to TBP. Values represent mean ± SE from three independent experiments. B. Cyclin D1 and Smad4 expression were determined by western blot. Tubulin was used as a loading control. Data are expressed as mean ± SE of fold change from PDGF stimulation from three independent experiments.

As noted, a major signalling pathway activated by TGFβ is phosphorylation of Smad2 and 3, which subsequently interact with Smad4 for translocation to the nucleus. To explore the possibility that Smad pathway mediates the TGFβ inhibition of PDGF-induced Cyclin D1 expression, VSMCs were transfected with siRNA against Smad4 (siSmad4) or siRNA Control (siNeg). Because Smads act as transcription factors, we first measured Cyclin D1 mRNA. VSMCs transfected with siSmad4 are no longer responsive to TGFβ and fail to exhibit a reduction in CyclinD1 mRNA when exposed to the combination of TGFβ and PDGF (Fig. 8A). Downregulation of Smad4 completely reverses the inhibitory effect of TGFβ on PDGF-stimulated CylinD1 protein expression as well (Fig. 8B). These data demonstrate that TGFβ inhibits the expression of Cyclin D1 mRNA expression induced by PDGF via Smad activation.

**Figure 9:**
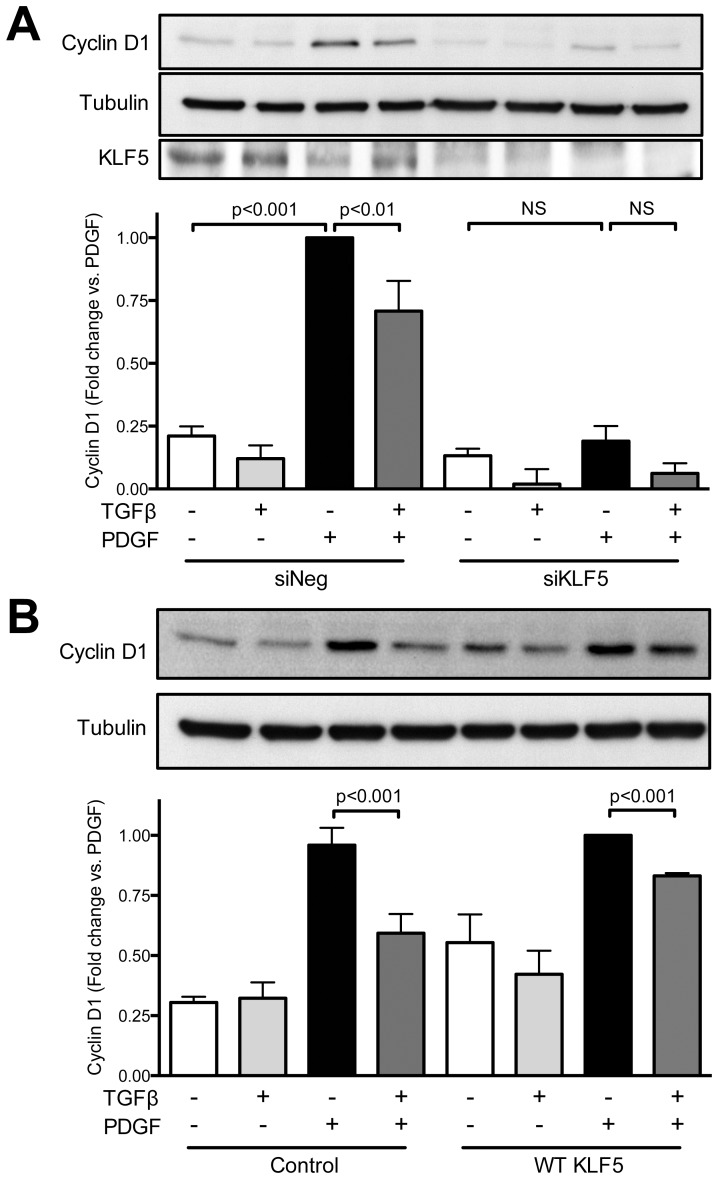
KLF5 is required for PDGF-induced upregulation of Cyclin D1 and mediates the inhibitory effect of TGFβ. A. VSMCs were transfected with control siRNA (siNeg) or siRNA against KLF5 (siKLF5) prior to stimulation with PDGF-BB, TGFβ or both as described in Fig 1. B. VSMCs were transduced with control vector or lentivirus expressing KLF5 prior to stimulation with PDGF-BB, TGFβ or both. Cyclin D1 and KLF5 expression were determined by western blot. Tubulin was used as a loading control. Data are normalized to tubulin and expressed as mean ± SE of fold change from PDGF stimulation from three independent experiments. P<0.001 for comparison of delta between PDGF alone and PDGF + TGFβ.

The Cyclin D1 promoter, however, does not contain a Smad binding element [23]. Based on the observation that both inhibition of proteasomal degradation and Smad4 siRNA completely inhibit TGFβ-induced Cyclin D1 downregulation, we hypothesized that a transcription factor regulated by Smad is the downstream effector of TGFβ. One such possibility is KLF5, which is regulated by Smad-associated ubiquitination [40] and has been implicated in Cyclin D1 upregulation [41]. We therefore tested a potential role for KLF5 in PDGF-induced Cyclin D1 upregulation and its inhibition by TGFβ. We found that siRNA against KLF5 abolished the increase in Cyclin D1 stimulated by PDGF and reduced Cyclin D1 levels in all conditions (Fig. 9A). Importantly, overexpression of KLF5 reversed the inhibitory effect of TGFβ on Cyclin D1 expression (Figure 9B). These data, together with the association of Smad pathways with KLF5 ubiquitination reported in the literature, strongly suggest that KLF5 is downstream of TGFβ-mediated, Smad-dependent downregulation of Cyclin D1.

**Figure 10:**
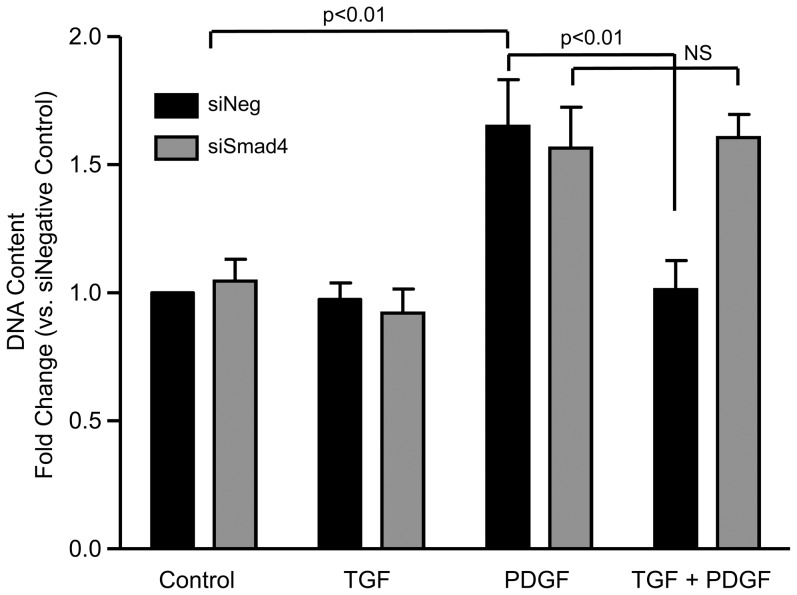
Smad4 is required for TGFβ-dependent inhibition of PDGF-induced proliferation. VSMCs were treated with PDGF-BB, TGFβ or both as described in Fig. 1 and DNA synthesis was measured using the fluorescent indicator CyQuant. Values represent mean ± SE from three independent experiments.

Finally, we analyzed the impact of the TGFβ-induced Smad/KLF5 pathway on the proliferation induced by PDGF. Consistent with the data shown in Fig. 1, we found that the increase in PDGF-induced proliferation is completely inhibited by TGFβ in siNeg transfected VSMC (1.05±0.1-fold increase in siNeg). Downregulation of Smad4 with siRNA reverses the inhibitory effect of TGFβ on PDGF-induced proliferation (1.6±0.1-fold increase in siSmad4) (Fig. 10). This result demonstrates that inhibition of the Smad pathway, and by inference KLF5, is functionally important in the antiproliferative effect of TGFβ.

## Discussion

Proliferation of VSMCs has been linked to the development and progression of several cardiovascular diseases such as atherosclerosis and restenosis. Here, we show for the first time that activation of the Smad pathway by TGFβ completely blocks PDGF-induced proliferation of VSMCs via inhibition of KLF5-mediated CyclinD1 expression. Many studies of proliferation in cell culture use a single agonist to investigate growth- or differentiation-related signalling pathways. However, the situation in vivo is complex, and cells are exposed to a multitude of agonists simultaneously. Our study underlines the importance of understanding how competing agonists simultaneously regulate cell phenotype and functional responses.

In this study, we used PDGF as a proliferative agent because previous work has demonstrated that PDGF is a critical mediator of VSMC growth and migration in vivo, as during restenosis [42]. Administration of neutralizing antibodies against PDGF or antisense oligonucleotide against PDGF receptor β reduce the intima thickening after balloon injury of carotid arteries [26], [27].

The effect of TGFβ on VSMC proliferation is more controversial. Several studies report a proliferative effect of TGFβ. Administration of oligonucleotides or antibodies against TGFβ has been demonstrated to reduce neointima formation after vascular injury [11], [43]. Moreover, Mao et al. [6] showed that VSMCs from smooth muscle specific Smad4 knockout mice are less proliferative. In the same study, the authors show that decreasing Smad2/3 using siRNA reduces the proliferation of VSMCs in response to serum. In contrast, others have described an antiproliferative effect of Smad activity induced by TGFβ in VSMCs. For example, Kobayashi et al. [13] demonstrated that Smad3 knockout mice, which have impaired TGFβ signalling, exhibit an increase in VSMC proliferation after vascular injury. The differential effect of TGFβ on growth does not seem to be due to confluence, as both subconfluent and confluent VSMC growth was inhibited by TGFβ (Fig. 1 and unpublished observations). This is in contrast to studies by Majack et al. [16], who found differences in the response to TGFβ depending on confluency. One explanation for this inconsistency might be the region of the aorta from which the cells were derived in each study, since aortic smooth muscle cells are of both mesenchymal and neural crest origin. Majack et al. did not indicate the origin of their cells, and we were unable to obtain this information from the commercial source. Our data are concordant with Kobayashi et al. and provide a potential explanation for these discrepancies. We suggest that the activation of the Smad pathway may be responsible for the antiproliferative effect of TGFβ, while non-Smad pathways could be pro-proliferative, so that depending on the relative activities of these signalling loops, the end result may be growth suppression or growth promotion. In support of this theory, Cohen et al. [15] demonstrated that TGFβ arrested EGF- or thrombin-treated VSMCs in the G1 phase of the cell cycle in an ERK1/2-independent manner. We found that TGFβ inhibits PDGF-induced proliferation in VSMCs via a Smad-dependent, Akt-independent pathway.

The cell cycle is complex process that involves multiple regulatory proteins including cyclins. Among them, Cyclin D1 is a key regulator for the progression of the cell cycle since its expression is required for the transition of G1 to S phase. Our results show that TGFβ inhibits PDGF-induced increases in Cyclin D1 mRNA expression. Moreover, we demonstrated that the Smad pathway is required for the inhibitory effect of TGFβ on Cyclin D1 mRNA expression induced by PDGF since downregulation of Smad4 by siRNA completely abolished the inhibitory effect of TGFβ. To our knowledge, this is the first report that links the activation of Smads to the inhibition of Cyclin D1 expression in VSMCs.

The exact mechanism by which Smad activation blocks Cyclin D1 expression appears to be via KLF5 degradation. While it remains possible that TGFβ reduces the expression of Cyclin D1 via direct repression by Smad, as has been established for the *c-myc* and *id1* genes [44], [45], this scenario is unlikely because the maximum phosphorylation (and therefore activation) of Smad occurs at 1 hour after TGFβ stimulation (unpublished observation), and the effect on Cyclin D1 is still evident after 24 hours of treatment. This suggests that inhibition may occur indirectly via an intermediate mediator. Our data indicate that TGFβ-induced Cyclin D1 downregulation is proteasome dependent, but that Cyclin D1 is not likely the direct target of the proteasome under these conditions (Fig. 7 and unpublished observations). Rather, it seems likely that TGFβ induces KLF5 degradation, thus interfering with the ability of PDGF to upregulate KLF5. This is supported by the observations that i) TGFβ inhibits PDGF-induced Cyclin D1 mRNA expression; ii) siKLF5 inhibits basal and PDGF-induced Cyclin D1 protein levels; and iii) the inhibitory effect of TGFβ is reversed by KLF5 overexpression. Of interest, Cordes et al. [46] and Long et al. [47] showed that TGFβ/Smad increases microRNA (miR) 145/143 [46], which reduces neointima thickening after vascular injury [48]. One of the well-described targets of miR145 is KLF5 [48]–[50], which is important in the expression of Cyclin D1 in VSMC [51], [52]. Whether microRNAs or proteasome-mediated degradation of KLF5 is the major target of TGFβ requires further study.

Additional insight into the upstream signals by which TGFβ regulates post-transcriptional processing of Cyclin D1 is also necessary. Diehl et al. [25] showed that inactivation of Glycogen Synthase 3β by Akt can prevent Cyclin D1 degradation via proteasomal processing. Interestingly, we observed that TGFβ stimulation reduces PDGF-induced Akt activity, which would be consistent with these observations. However, Fig. 6 suggests that although inactivation of Akt by TGFβ could theoretically decrease the stability of Cyclin D1 via proteasome degradation, it is not likely the case here since TGFβ is still able to inhibit Cyclin D1 expression even in the presence of constitutively active Akt. Our results suggest that when PDGF and TGFβ are present together, the role of the proteasome in Cyclin D1 processing is not direct, as Diehl et al. showed, but rather is likely the consequence of proteasomal targeting of a transcription factor such as KLF5.

In summary, we found that (i) TGFβ inhibits PGDF-induced proliferation in human VSMCs; (ii) TGFβ reduces the transcription of PDGF-induced Cyclin D1 via KLF5; (iii) inhibition of proteasome degradation also reverses TGFβ-mediated downregulation of Cyclin D1, presumably by preventing KLF5 ubiquitination; iv) Smad4 is critical for the inhibition of Cyclin D1 expression induced by TGFβ most likely by regulating KLF5; and (iv) Smad4 is required for the antiproliferative effect of TGFβ. Since previous reports have shown that proliferation of VSMCs plays a crucial role in the progression of several cardiovascular diseases such as restenosis and arteriosclerosis, a better understanding of this response, and the interactions between pro- and anti-proliferative agonists, could ultimately lead to novel therapeutic targets for treatment of these diseases.
